# Cell-free synthesis of stable isotope-labeled internal standards for targeted quantitative proteomics

**DOI:** 10.1016/j.synbio.2018.02.004

**Published:** 2018-02-21

**Authors:** Ryohei Narumi, Keiko Masuda, Takeshi Tomonaga, Jun Adachi, Hiroki R. Ueda, Yoshihiro Shimizu

**Affiliations:** a Laboratory of Proteome Research, National Institutes of Biomedical Innovation, Health and Nutrition, 7-6-8, Satio-Asagi, Ibaraki, Osaka 567-0085, Japan; b Laboratory for Single Cell Mass Spectrometry, RIKEN Quantitative Biology Center (QBiC), 6-2-3, Furuedai, Suita, Osaka 565-0874, Japan; c Department of Systems Pharmacology, Graduate School of Medicine, The University of Tokyo, 7-3-1 Hongo, Bunkyo-ku, Tokyo 113-0033, Japan; d Laboratory for Synthetic Biology, RIKEN Quantitative Biology Center (QBiC), 6-2-3, Furuedai, Suita, Osaka 565-0874, Japan; e Laboratory for Cell-Free Protein Synthesis, RIKEN Quantitative Biology Center (QBiC), 6-2-3, Furuedai, Suita, Osaka 565-0874, Japan

**Keywords:** Absolute quantification, Mass spectrometry, Cell-free protein synthesis system, In vitro translation, Targeted quantitative proteomics, PURE system

## Abstract

High-sensitivity mass spectrometry approaches using selected reaction monitoring (SRM) or multiple reaction monitoring (MRM) methods are powerful tools for targeted quantitative proteomics-based investigation of dynamics in specific biological systems. Both high-sensitivity detection of low-abundance proteins and their quantification using this technique employ stable isotope-labeled peptide internal standards. Currently, there are various ways for preparing standards, including chemical peptide synthesis, cellular protein expression, and cell-free protein or peptide synthesis. Cell-free protein synthesis (CFPS) or in vitro translation (IVT) systems in particular provide high-throughput and low-cost preparation methods, and various cell types and reconstituted forms are now commercially available. Herein, we review the use of such systems for precise and reliable protein quantification.

## 1 Introduction

Cells survive by constituting very complicated molecular networks involving various small molecules and macromolecules including nucleic acids, proteins, carbohydrates and lipids. A wide variety of complex biological functions such as migration, predation and metabolism are orchestrated by functioning networks that contribute to prevent entropy from increasing in non-equilibrium systems, as discussed by Schrödinger [1], which essentially forms the basis of all life activities. While individual reactions in a network operate via simple mechanisms governed by thermodynamics, complexity is generated by the enormous number of reactions in the network. Therefore, the most intuitive way to understand this complexity is to accurately measure the information present among the individual molecules over time, and to clarify the characteristics of the entire reaction network by data assimilation with the model of an entire cellular network [2] or a partial molecular network [3]. Among the molecules involved, proteins, the expression products of genetic information, are the major cellular components responsible for almost all biochemical processes. Thus, a systems-level biological approach based on quantitative proteomics [4] is crucial for understanding life.

Traditionally, quantification of proteins has been conducted using radioactive isotopes or antigen-antibody interactions. Recently, methods using intracellular detection of protein dynamics by fluorescence or luminescence imaging coupled with genetic engineering have also been adopted [[5], [6], [7]]. However, for detecting and quantifying proteins as they occur in biological samples without using special procedures employing antibodies and/or genetic engineering, mass spectrometry (MS) is most suitable method, in terms of both throughput and sensitivity. MS-based quantitative global proteomics researches have revealed whole proteome maps of yeast, mouse and human cells [[8], [9], [10], [11], [12], [13]], detecting more than 10,000 proteins in the process, demonstrating its high-throughput capabilities.

In standard liquid chromatography (LC)-MS approaches, biological samples are processed with proteases such as trypsin, which cleaves peptide bonds between the carboxyl group of arginine or lysine and the amino group of the adjacent amino acid. The resultant proteotypic peptides, specific to their parent proteins, are separated by LC and individually subjected to MS analysis, which separates each peptide peak into two dimensions based on their individual chemical properties and masses. The addition of a pre-fractionation step such as strong cation exchange (SCX) column chromatography can further increase the sensitivity by allowing three- or more-dimensional separation [14,15]. The introduction of tandem mass spectrometry, also termed MS/MS, can improve the detection resolution by determining the peptide sequences through the generation of fragment ions from precursor ions. MS/MS is an indispensable technique for both global and targeted proteomics. Sequence determination by MS/MS is necessary for shotgun analysis of various peptides in global proteomics approaches. For selected reaction monitoring (SRM) or multiple reaction monitoring (MRM) methods that are frequently employed in targeted proteomics experiments, selection of both precursor and fragment ions by a triple quadrupole mass spectrometer is necessary to limit the number of peptides observed for each target, which enables highly sensitive analysis of target peptides in samples.

**Fig. 1:**
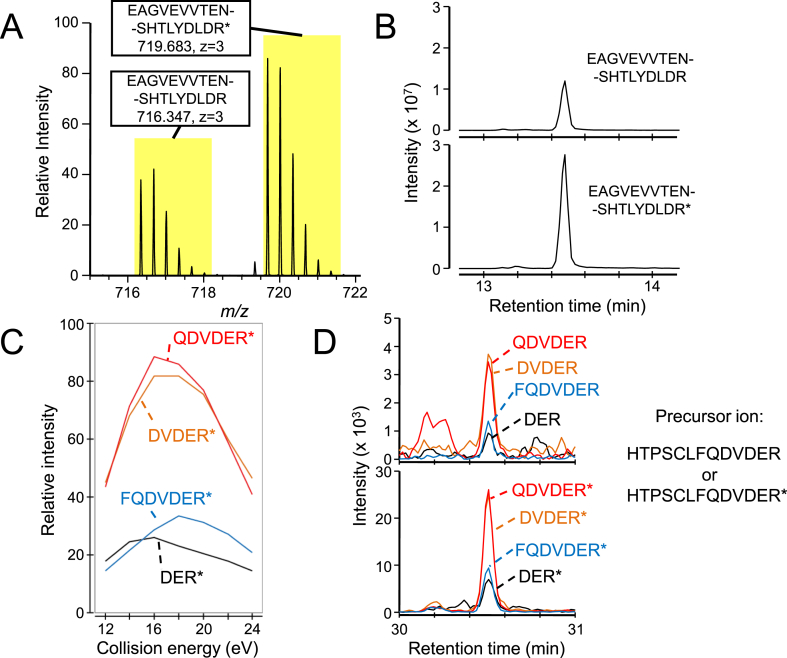
Quantification of peptides in samples using stable isotope-labeled peptide internal standards. (A) and (B) LC-MS analysis of the EAGVEVVTENSHTLYDLDR peptide. Mass spectrum (A) and mass chromatograms (B) of a peptide in the sample with a trivalent ion valency (*m*/*z* = 716.347) and its isotope-labeled form synthesized for quantification (*m*/*z* = 719.683). The retention time of the two peptides is identical, and the quantification can be performed by comparison of the peak areas in each mass chromatogram. (C) and (D) SRM analysis of the HTPSCLFQDVDER peptide. In this case, transitions of four fragment ions (FQDVDER, QDVDER, DVDER and DER) from the precursor ion (HTPSCLFQDVDER) are monitored. The collision energy for each transition is optimized using the synthesized isotope-labeled peptide (C), and both transitions from the non-labeled and labeled precursor ions are monitored (D). Quantification can be performed by comparison of peak areas in each SRM transition. The asterisk (*) indicates the isotope-labeled amino acid. MS analyses were performed according to the previous study [69].

Since the intensity of peptide peaks depends on their physical properties, absolute quantification of peptides of interest by MS is achieved by utilizing a stable isotope-labeled peptide internal standard with the same sequence at a known concentration [16]. Apart from the difference in mass, all other chemical and physical properties of the two peptides are identical; hence retention times in LC, ionization efficiencies and fragmentation patterns in MS/MS are all identical. Thus, quantitative determination of peptides of interest can be carried out only by comparing the intensities of the two peaks (Fig. 1). It is over three decades since this concept was first proposed [17], and it is now possible to prepare peptide standards in various ways. In recent years, targeted quantitative proteomics using internal standards synthesized by cell-free protein synthesis (CFPS) or in vitro translation (IVT) systems have gradually attracted attention. This review focuses on the cell-free synthesis of stable isotope-labeled internal standards for targeted quantitative proteomics studies.

## 2 Chemical peptide synthesis and cellular protein synthesis

**Fig. 2:**
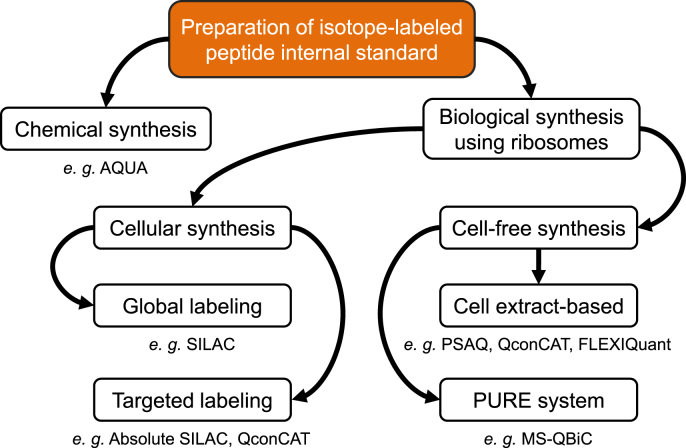
Approaches currently employed for preparing stable isotope-labeled peptide internal standards.

Various approaches currently employed for preparing stable isotope-labeled peptides are shown in Fig. 2. The methods can be roughly divided into two types, chemical synthesis from the C-terminus, and biological synthesis from the N-terminus using ribosomes, among which chemically synthesized peptides represented by Absolute Quantification (AQUA) is the most widely used method [18]. AQUA peptides are generated by solid-phase synthesis [19], in which peptides bound to a solid matrix with a free N-terminal amino group are reacted with an N-terminally protected amino acid, followed by deprotection of the amino group and washing of the solid phase. Peptides can be elongated at their N-terminus by consecutive reactions. Stable isotope labels can be introduced by replacing one residue with a stable isotope-labeled amino acid. Moreover, by using modified amino acid such as phosphorylated serine as a substrate, modified peptides that mimic post-translational modification can be produced, demonstrating the versatility of this approach.

However, there are some limitations associated with this method. It requires large amounts of isotopically labeled amino acids as substrates. Synthesis can be difficult depending on the peptide sequence due to sequence-dependent side reactions. These limitations make it difficult to reduce the cost per peptide, which is a barrier to multiplexed synthesis. To overcome these drawbacks, multiplexed chemical peptide synthesis, termed SPOT synthesis [20], is applied to establish SRM assays in a high-throughput manner using crude (impure) peptides without laborious purification [21]. A stable isotope dimethyl labeling method [22,23] where the chemically synthesized peptides are labeled with stable isotope-labeled formaldehyde is another cost-effective alternative for MS-based quantification [24,25].

Biological synthesis can be further divided into cellular and cell-free synthesis approaches (Fig. 2). Stable Isotope Labeling by Amino acids in Cell culture (SILAC) is a representative cellular synthesis method [26]. In this method, the composition of the medium is adjusted so that a stable isotope-labeled essential amino acid is necessarily introduced into the protein as it is, so that a specific amino acid of the intracellular protein is completely replaced with a labeled residue. Unlike chemical synthesis methods, stable isotope labeling can be introduced nonspecifically to intact cell proteins, enabling various peptides to be synthesized simultaneously, which is more effective for global proteomics analysis. In recent years, SILAC-based global quantitative proteomics has been applied at the tissue, organism and individual level by growing on a diet containing isotope-labeled amino acids [[27], [28], [29], [30], [31]].

Development towards targeted quantitative proteomics has also progressed. In the Absolute SILAC method, targeted analysis can be carried out by constructing expression systems for specific proteins in cells, and tryptic digests of the produced proteins are used as internal standards [32]. Cellular protein expression systems are also used in the QconCAT method, which uses tryptic digests of artificial proteins comprising a concatenation of proteotypic peptides [33,34].

## 3 Cell-free protein synthesis systems

In general, to prepare a specific protein sample, a recombinant protein expression system is employed in which the gene encoding the protein of interest is inserted into an expression vector and protein is produced in *Escherichia coli*, yeast or other cells. Once the method is established, it has an advantage that the target protein samples can be obtained reproducibly, and therefore, it is a method indispensable for preparation of protein samples. However, to perform protein production using this system, it is necessary to go through laborious processes such as DNA cloning, cell transformation, culturing and disruption, and protein purification from cell lysates. Additionally, proteins that are toxic to host cells are difficult to produce using this approach.

CFPS and IVT methods have been developed to replace cellular protein production systems [[35], [36], [37], [38]]. These systems are not limited by the need to maintain living organisms, and can therefore synthesize proteins that are toxic to cells. Proteins can be synthesized only by adding template DNA or RNA in a high-throughput manner. Artificial manipulation of the system is easy; hence proteins containing isotope-labeled amino acids or non-natural amino acids can be synthesized by changing the components of the system. Thus, there are many advantages compared with protein expression systems using living cells.

**Fig. 3:**
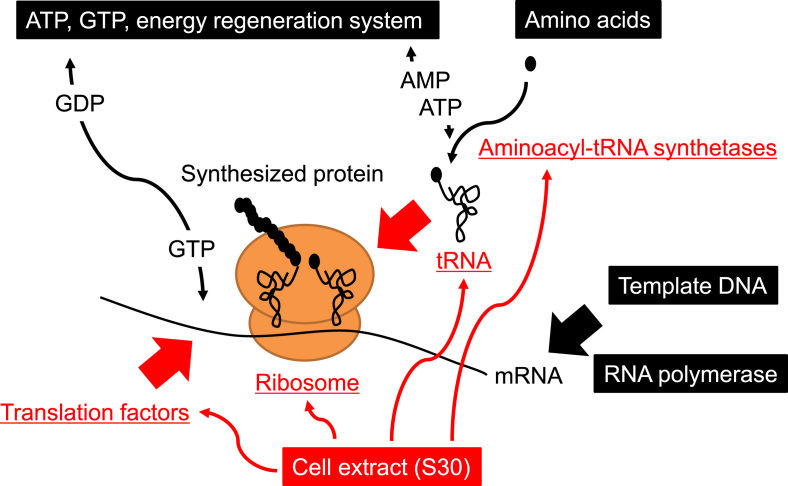
Cell-free protein synthesis systems based on the S30 cell extract. Ribosomes, translation factors, tRNA and aminoacyl tRNA synthetases necessary for protein synthesis are supplied by the S30 extract. Template mRNA is synthesized in the same reaction mixture by introducing template DNA and RNA polymerase into the system. Efficient protein synthesis is performed by adding substrate amino acids, ATP, GTP and an energy regeneration system.

The CFPS/IVT system was originally developed by Nirenberg and Matthaei [39] for polyphenylalanine synthesis by adding polyuridylic acid to a cell extract obtained from *E. coli* cells. This experiment revealed that UUU (TTT in the DNA sequence) encodes phenylalanine in the genetic code. This was also the first example of template-dependent synthesis of a polypeptide with a specific amino acid sequence, which underpinned the subsequent development of CFPS/IVT systems. The prototype of currently commercially available CFPS/IVT systems, in which template mRNA transcription and protein synthesis is performed simultaneously in the same reaction mixture, was developed by Zubay et al. [40]. Using a cell extract termed S30, which represents a supernatant derived from centrifugation at 30,000 *g*, RNA polymerases, transcription substrates (ATP, GTP, CTP and UTP), translation substrates (amino acids), and energy substrates such as creatine phosphates and phosphoenolpyruvates are mixed, and template DNA is added to initiate transcription and translation (Fig. 3). For synthesis of internal standards for quantitative proteomics using CFPS/IVT, it is easy to use stable isotope-labeled amino acids as a substitute for non-labeled one where their concentration is sufficient under millimolar range. Furthermore, the reaction volume can be scaled from several microliters to several tens of microliters, making the method extremely cost-effective.

Methods employing cellular protein expression systems such as Absolute SILAC [32] and QconCAT [33,41,42] can also adopt CFPS/IVT systems to take advantage of these benefits. The Protein Standard Absolute Quantification (PSAQ) method, similar to Absolute SILAC but specialized for CFPS/IVT use, has also been developed for targeted quantitative proteomics [43,44]. In these methods, intact proteins or artificial proteins comprising a concatenation of proteotypic peptides are expressed using *E. coli* cell extracts in the presence of a stable isotope-labeled amino acid. The products are subjected to tryptic digestion and analyzed simultaneously with samples of interest, as occurs in methods using cellular protein expression systems. It should be noted that, in methods using cellular expression systems, the homogeneity of isotope-labeled peptides is reduced due to conversion of labeled amino acids into other molecules, or *vice versa*, by cellular metabolic enzymes, which leads to inaccuracies in quantification [32,45]. Although commercially available CFPS/IVT kits can be used in proteomics studies, a similar problem can also occur when using CFPS/IVT systems [46,47]. Ideally, systems should be constructed using cell extracts prepared from strains deficient in specific metabolic enzymes such as those employed in Absolute SILAC [32], but where this is impractical or impossible, the degree of amino acid conversion in the MS analysis should be carefully considered.

One major advantage of CFPS/IVT systems is the large number of reactions that can be performed in a small reaction volume, and various studies have capitalized on this in recent years. CFPS/IVT-based high-throughput protein synthesis is performed to efficiently select highly sensitive proteotypic peptides necessary for establishing the SRM/MRM method [48]. Other studies also used CFPS/IVT systems for selecting sensitive proteotypic peptides to detect low-abundance proteins such as transcription factors [49] or “missing” proteins not detected by global proteomics studies [50]. Thus, approaches using CFPS/IVT systems are regularly adopted for high-sensitivity analysis using SRM/MRM.

CFPS/IVT systems can also use cell extracts from organisms other than *E. coli*, and systems based on archaeal, protozoan, fungal, plant, insect and mammalian cell extracts have been reported [38]. Among them, a wheat germ cell extract system is now widely used for quantitative proteomics research on eukaryotic proteins due to its high stability and translation efficiency. As originally reported, wheat germ embryos are washed several times to prepare cell extract solutions in which endosperm-derived enzymes that inhibit the translation system are removed [51]. Template DNA amplified by PCR can be used for transcription and translation coupled systems, and it is now possible to conduct high-throughput proteomics research using the wheat germ system [52].

Several reports using the wheat germ system for quantitative targeted proteomics have been reported. In the Full-Length Expressed Stable Isotope-labeled Proteins for Quantification (FLEXIQuant) method, full-length eukaryotic proteins are expressed in the wheat germ system, directly labeled with a stable isotope-labeled amino acid and used for MS quantitative analysis of post-translational modifications [53]. Using this strategy, a workflow was developed to monitor quantitative information about protein phosphorylation (FLEXIQinase) [54]. A similar approach was also utilized in a quantitative study of transmembrane proteins where 263 proteins were analyzed from cDNA clones [55]. The same group also integrated the QconCAT method with the wheat germ system to prepare a reference stable isotope-labeled peptide library for the development of a large-scale SRM/MRM assay for targeted proteomics [56]. Using mTRAQ labeling [57], which originated from Isobaric Tags for Relative and Absolute Quantification (iTRAQ) labeling [58], rather than direct labeling using a stable isotope-labeled amino acid, over 18,000 human recombinant proteins were expressed form cDNAs using the wheat germ system, and used to develop experimentally verified MRM assays of mass tag-labeled peptides, resulting in the In vitro Proteome-assisted Multiple Reaction Monitoring for Protein Absolute Quantification (iMPAQT) platform [59].

## 4 The protein synthesis Using Recombinant Elements (PURE) system for direct cell-free peptide synthesis

CFPS/IVT systems using the S30 cell extract contain all the necessary elements for protein synthesis, such as ribosomes, tRNA, translation factors and aminoacyl tRNA synthetases (Fig. 3). However, these systems also contain protein factors or enzymes unrelated to the protein synthesis process that can make fine control difficult because it is impossible to accurately grasp what their impact may be, and what kind of reactions are occurring in the reaction mixtures. To overcome this disadvantage, the Protein synthesis Using Recombinant Elements (PURE) system, a reconstituted CFPS system from individual components, was constructed [60]. Unlike the S30-based system, the components included in the PURE system are known, and their exact composition is controllable. Additionally, stable protein synthesis can be performed since factors or enzymes that are inhibitory to the translation system, such as nucleases or proteases, are much less abundant [61]. Furthermore, like cell extract-based systems, this system is commercially available, but the protocol for preparation is also in the public domain, allowing researchers to prepare it by themselves [62,63]. More simplified preparation method that enables co-purification of PURE system components by inserting hexa-histidine sequences into these genes by applying Multiplex Automated Genome Engineering (MAGE) [64,65], by *de novo* designing large cistron consists of these genes [66], and by constructing synthetic microbial consortia [67] are also developed and available.

**Fig. 4:**
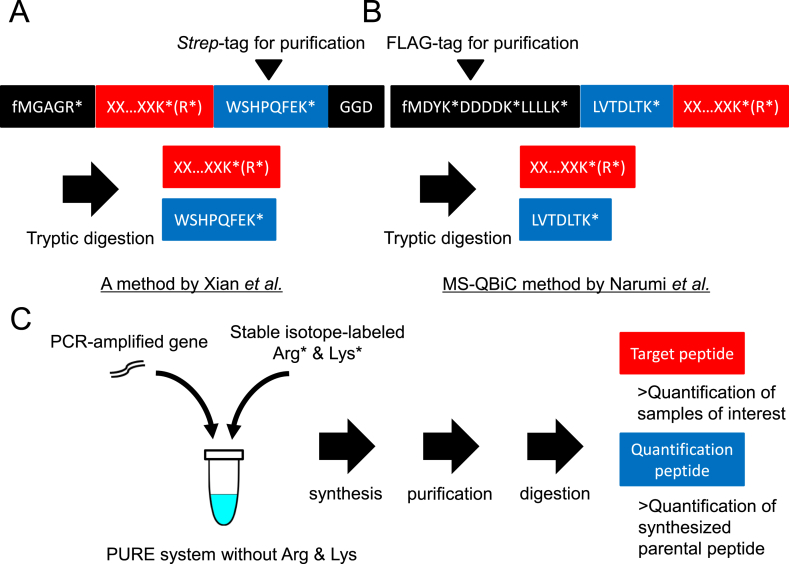
The PURE system for targeted quantitative proteomics studies. (A) Peptide synthesis using the method of Xian et al. [68]. A target peptide ending with a stable isotope-labeled arginine or lysine (colored red) and a fixed peptide with the sequence WSHPQFEK* (colored in blue) are produced by tryptic digestion. (B) Peptide synthesis using the MS-QBiC method of Narumi et al. [69]. A target peptide ending with a stable isotope-labeled arginine or lysine (colored red) and a fixed peptide with the sequence LVTDLTK* (colored blue) are produced by tryptic digestion. (C) Typical schemes for both methods. PCR-amplified DNA template for peptide synthesis and stable isotope-labeled arginine and lysine are added to the PURE system without non-labeled arginine and lysine to synthesize the parental peptide. The peptide is affinity-purified, and target and quantification peptides are produced by tryptic digestion. The quantification peptide is used for quantification of the synthesized parental peptide, and the target peptide is used for quantification of the sample of interest. The character ‘f’ indicates the formyl group of the synthesized peptide, and the asterisk (*) indicates the isotope-labeled amino acid.

Recently, two research groups reported that features of the PURE system can be utilized for targeted quantitative proteomics studies [68,69]. Both schemes are similar but different in detail, synthesizing a short peptide in the form of a purification tag and a target peptide concatenated. Xian et al. [68] synthesized a peptide in which a fixed five-residue peptide (MGAGR), a variable target peptide and a fixed 11-residue peptide (WSHPQFEKGGD), including *Strep*-tag (WSHPQFEK) for purification and quantification, are concatenated in this order (Fig. 4A). In the MS-Quantification By isotope-labeled Cell-free products (MS-QBiC) workflow reported by Narumi et al. [69], a peptide with a fixed FLAG-tag with an N-terminal methionine (MDYKDDDDK) for purification, a fixed five-residue spacer peptide (LLLLK), a fixed seven-residue peptide (LVTDLTK) derived from bovine serum albumin (BSA) for quantification and a variable target peptide are concatenated in this order (Fig. 4B).

In both cases, a PCR-amplified DNA template is used for peptide synthesis using the PURE system (Fig. 4C). The template contains all elements required for peptide synthesis using an *E. coli*-based translation system, including a T7 promoter, a Shine-Dalgarno sequence and an open reading frame. PCR amplification is completed within two steps, and amplified DNA is directly used in the PURE system where the translation reaction is carried out in the presence of isotope-labeled arginine and lysine (Fig. 4C). The synthesized peptide is purified using magnetic beads and used for LC-MS or SRM/MRM analyses. For quantification of the synthesized peptide, spiking with unlabeled *Strep*-tag peptide (WSHPQFEK) or quantification peptide (LVTDLTK) and tryptic digestion are performed to separate the parental peptide into individual peptides. In the case of MS-QBiC, spiking of full-length BSA at a known concentration can also be utilized. The determined concentration of the synthesized peptide is used for quantification of target peptides in the sample of interest (Fig. 4C).

Three features arising from the fact that the PURE system is a reconstituted system are exploited in these studies. Firstly, contamination of metabolic enzymes is highly suppressed, and the homogeneity of isotope-labeled peptides is therefore ensured, resulting in reliable quantification. Secondly, DNA degradation by nucleases is unlikely to occur, which enables the use of very short PCR-amplified DNA templates for peptide expression. It is not necessary to perform the laborious cDNA cloning or gene synthesis required for conventional methods employing cellular or cell extract-based protein expression systems. Peptide synthesis can be achieved only by designing appropriate oligo DNA and performing PCR, which enables immediate synthesis of peptides conducive to high-throughput experiments. Thirdly, DNA, RNA and peptide degradation by nucleases and proteases is unlikely to occur; hence stoichiometric equivalence of quantification peptides and target peptides in the synthesized parent peptide is guaranteed. When linear DNA templates are used in cell extract-based systems, DNA and transcribed RNA can be severely degraded, and various precautions must be taken in terms of template sequence or the use of specific strains for preparing cell extracts [[70], [71], [72]]. In addition, it is difficult to prepare peptides using cell extract-based systems because it is not possible to exclude peptidases such as oligopeptidase [73]. By contrast, the parental peptide is intact in the PURE system, and the molar ratio between quantification peptides and target peptides is preserved, enabling a more reliable quantification.

High-throughput peptide synthesis using the PURE system is exploited in the MS-QBiC workflow. The developed scheme has been applied to the quantification of 20 circadian clock proteins using 120 target peptides via SRM/MRM analysis. Using 47 peptides with a signal-to-noise ratio sufficient for the detection of endogenous targets, the circadian dynamics of 16 proteins expressed in the mouse liver were illuminated [69].

## 5 Future perspectives for obtaining more peptides for high-sensitivity quantification

Analysis of the expression dynamics of circadian clock proteins using the MS-QBiC workflow [69] demonstrates the necessity to prepare and synthesize as many peptides as possible from parental proteins. Despite the successful synthesis of numerous designed peptides, more than half (73/120) were not suitable for quantification, mainly due to their low signal-to-noise ratio for the detection of target proteins. Thus, because theoretically designed peptides are often unsuitable for quantification due to their physical properties, it is desirable to prepare as many peptides for each target protein as possible, which is one of the reasons why CFPS/IVT systems are useful for proteomics studies. In addition, a variety of factors, including the presence of unknown protein isoforms and unknown post-translational modifications, and inefficient or missed cleavages by tryptic digestions, can affect the precise quantification of proteins. To overcome this problem, it is important to use many peptides for the analysis of each target protein.

It is also necessary to improve data interpretation. Because the preparation of many peptide candidates as internal standards has been difficult, a generalized scheme for quantifying one protein with multiple peptides has not yet been proposed. While some reports do not discuss the correctness of the obtained quantitative values, others do address this issue. For example, a recent study, in which over 1800 *Saccharomyces cerevisiae* proteins were absolutely quantified using the QconCAT method [74], did not include peptides with low quantitative values based on certain criteria. However, there are currently no clear criteria for selection, and it is necessary to establish a standard method for uniform protein quantification. Detailed analyses using many peptides for each target protein are also important for this purpose.

Although trypsin-based protein cleavage is currently considered the gold standard in the field of MS-based proteomics [75], a major problem arises because it is difficult to obtain a large number of candidate peptides for internal standards, especially for basic proteins containing many Lys and Arg residues. Although other proteases such as chymotrypsin and glutamyl endopeptidase are also used in some cases [76,77], it is important to explore other proteases that are versatile in terms of specificity and efficiency. Increasing the number of internal standards obtained for each target protein can be achieved using new proteases via metagenomics [78,79] or by designing *de novo* proteases via computational methods [80]. Such approaches are likely to lead to more accurate and quantitative proteomics approaches in the future.

## Declaration of interest

None.
